# Determination of total arsenic and arsenic species in drinking water, surface water, wastewater, and snow from Wielkopolska, Kujawy-Pomerania, and Lower Silesia provinces, Poland

**DOI:** 10.1007/s10661-016-5477-y

**Published:** 2016-08-04

**Authors:** Izabela Komorowicz, Danuta Barałkiewicz

**Affiliations:** Department of Trace Element Analysis by Spectroscopy Method, Faculty of Chemistry, Adam Mickiewicz University in Poznań, 89b Umultowska Street, 61-614 Poznań, Poland

**Keywords:** Total arsenic, Arsenic species, Speciation, Water, ICP-MS, HPLC/ICP-MS

## Abstract

Arsenic is a ubiquitous element which may be found in surface water, groundwater, and drinking water. In higher concentrations, this element is considered genotoxic and carcinogenic; thus, its level must be strictly controlled. We investigated the concentration of total arsenic and arsenic species: As(III), As(V), MMA, DMA, and AsB in drinking water, surface water, wastewater, and snow collected from the provinces of Wielkopolska, Kujawy-Pomerania, and Lower Silesia (Poland). The total arsenic was analyzed by inductively coupled plasma mass spectrometry (ICP-MS), and arsenic species were analyzed with use of high-performance liquid chromatography inductively coupled plasma mass spectrometry (HPLC/ICP-MS). Obtained results revealed that maximum total arsenic concentration determined in drinking water samples was equal to 1.01 μg L^−1^. The highest concentration of total arsenic in surface water, equal to 3778 μg L^−1^ was determined in Trująca Stream situated in the area affected by geogenic arsenic contamination. Total arsenic concentration in wastewater samples was comparable to those determined in drinking water samples. However, significantly higher arsenic concentration, equal to 83.1 ± 5.9 μg L^−1^, was found in a snow sample collected in Legnica. As(V) was present in all of the investigated samples, and in most of them, it was the sole species observed. However, in snow sample collected in Legnica, more than 97 % of the determined concentration, amounting to 81 ± 11 μg L^−1^, was in the form of As(III), the most toxic arsenic species.

## Introduction

Arsenic is widely distributed in surface water, groundwater, and drinking water. Its concentration in different types of water varies considerably. In some cases, it significantly exceeds expected mean values for arsenic and maximum permissible arsenic concentration allowed for drinking water, indicating a degree of pollution (Fowler et al. 2007).

Arsenic pollution is a worldwide problem many scientists have repeatedly expressed concern about. As a result, the biological and environmental consequences of its contamination are being studied in detail. Although most researchers focus on the arsenic originating from the natural sources, human activities (such as smelting of arsenic bearing minerals, the disposal of industrial waste, or burning of fossil fuels) can locally introduce a very high contamination (Bissen and Frimmel 2003; Matschullat 2000).

Issue of great importance is presence of arsenic in groundwater used as a source of drinking water. In recent years, cases of arsenic pollution have been reported in many countries such as the USA, China, Bangladesh, Pakistan, Taiwan, Chile, Argentina, Japan, Turkey, Thailand, Mexico, Vietnam, and India (Simsek 2013; He and Charlet 2013; Sorg et al. 2014; Smedley and Kinniburgh 2002; Berg et al. 2001; Gammons et al. 2005, Brahman et al. 2013). Due to permanent excessive level of arsenic, some countries, including Bangladesh, Mexico, Vietnam, and India, have considerably raised its level in drinking water. It is presumed that around 40 million people in Bangladesh live at immediate risk due to arsenic pollution (Bissen and Frimmel 2003). An analysis of 20,000 tube-well waters indicated that arsenic levels in drinking water are above the maximum permissible concentration limit of 10 μg L^−1^ (WHO 2011) in case of 62 % of tube-well waters. In some places, its concentration was as high as 3700 μg L^−1^ (Bagla and Kaiser 1996). There are also many papers reporting similar arsenic concentration, reaching several thousand μg L^−1^ (Berg et al. 2001; Brahman et al. 2013; Smedley et al. 2002; Farnfield et al. 2012; Gammons et al. 2005; Aiuppa et al. 2003; Bednar et al. 2004). It has been estimated that as many as 60–100 million people globally may be at risk of exposure to excessive levels of arsenic (Ng et al. 2003). Since arsenic contamination provides such a huge problem in many places in the world, it is worth to mention that various methods of its elimination from the aqueous environments are available. Besides conventional remediation techniques such as osmosis, ion exchange, or electrodialysis, for removing metal or metalloids from water, biosorption is characterized by the significant development in recent years [Basu et al. 2014; Abid et al. 2016]. One of the greatest advantages of this process is material of biosorbent, which is easy and commonly available, and inexpensive. A lot of different agricultural and food-industry biowastes such as coconut shell, coconut coir pith, mango leaf, rice polish, or tea waste have been examined as potential biosorbents for As-contaminated water. These various approaches are widely discussed in the paper of Shakoor et al. (2016). One of the most recent paper concerns As(V) biosorption with application of food processing biowastes, more specifically orange peel biowaste. This research revealed that with use of charged orange peel, it was possible to remove 98 % of As(V) from the solution containing 200 mg L^−1^ of As(V) using 4 g L^−1^ of biosorbent [Abid et al. 2016].

In most aquifers, bio-geo interactions probably dominate as the source of arsenic. Interaction of arsenic with organic and mineral colloids can elevate its concentration (He and Charlet 2013). Arsenic can exist in water in several organic and inorganic forms. Speciation of arsenic depends on pH, salinity, acid dissociation constant at logarithmic scale (pK_a_), and redox potential (Sugár et al. 2013). The toxicity of arsenic compounds decreases in the following order: arsines > inorganic arsenites > organic trivalent compounds (arsenooxides) > inorganic arsenates > organic pentavalent compounds > arsonium compounds > elemental arsenic (Adriano 2001; Fowler 1983; Mandal and Suzuki 2002). Arsenobetaine and arsenocholine are considered as nontoxic (Komorowicz and Barałkiewicz 2011).

It is well known that toxicological and environmental impact of arsenic strictly depends on the chemical form of this element (Cornelis et al. 2005; Brahman et al. 2013). Although there is a vast amount of information on the occurrence and concentration of total arsenic in different types of water, the data on the speciation of arsenic are limited (Sorg et al. 2014; Haque and Johannesson 2006). The shortage of information on the speciation of arsenic may be partially attributed to the complexity, cost, and time needed to perform arsenic speciation analyses (Sorg et al. 2014). Nevertheless, it is essential to develop sensitive and precise analytical procedures to identify and quantify arsenic species in water (Baig et al. 2010; Hirata and Toshimitsu 2005).

The USEPA has elaborated a document reviewing the science and technologies for monitoring arsenic in the environment (USEPA 2004). The methods approved by USEPA include inductively coupled plasma-mass spectrometry (ICP-MS), inductively coupled plasma atomic emission spectrometry (ICP-AES), graphite furnace atomic absorption (GFAAS), and hydride generation atomic absorption spectrometry (HGAAS), all of which may be characterized by method detection limits (MDL) ranging from 0.5 to 50 μg L^−1^ (USEPA 1999; Ma et al. 2014). The choice of an adequate analytical method is dictated by the purpose of our analysis, the level of analyte’s concentration in concrete matrix, and the type of compound in which analyte is present. The most often applied strategy is connection of HPLC separation with ICP-MS detection (Komorowicz and Baralkiewicz 2014; Bednar et al. 2004; Shraim et al. 2002; Deng et al. 2009; Jabłońska-Czapla et al. 2014) or AFS detection (Yu et al. 2014; Gong et al. 2006; Keller et al. 2014; Farias et al. 2015). Coupling of HG system to AAS (Berg et al. 2001; Affum et al. 2015); ICP-MS (Musil et al. 2014), ASF (Aiuppa et al. 2003; Deng et al. 2009; Musil et al. 2014), or ICP-AES (Smedley et al. 2002) is also quite popular. There are either reports about application of SPE system used for separation purposes before detection by WDXRF, ICP-MS, DLLME-SFO, GF-AAS, and ETAAS (Brahman et al. 2013; Baig et al. 2010; Shamsipur et al. 2014; Peng et al. 2015; Hagiwara et al. 2015).

Although a variety of analytical techniques have been already applied for arsenic species determination, as mentioned, HPLC separation which is followed by ICP-MS or HG-AFS undeniably belong to the most often used hyphenated techniques. ICP-MS technique is most commonly applied for speciation analysis, and this tendency is expected to continue to grow as the required instrumentation becomes more widely available. Said method provides reliable quantitative data for arsenic species at environmentally relevant levels in diverse matrices (Ma et al. 2014) as well as other significant advantages. These include the excellent separating power of HPLC, associated with a high degree of element specificity and very low detection limits of the ICP-MS technique. HPLC/ICP-MS can detect arsenic in inorganic and organic forms with high precision. Additionally, this system is totally integrated and automated (Liévremont et al. 2009; Tomlinson et al. 1995).

The objective of this study was to determine total arsenic and arsenic species concentration in drinking water, surface water, wastewater, and snow samples collected from Wielkopolska, Kujawy-Pomerania, and Lower Silesia provinces located in Poland. The last mentioned province is the As-affected area known for geogenic contamination with this element.

## Experimental

### Sampling sites

**Fig. 1:**
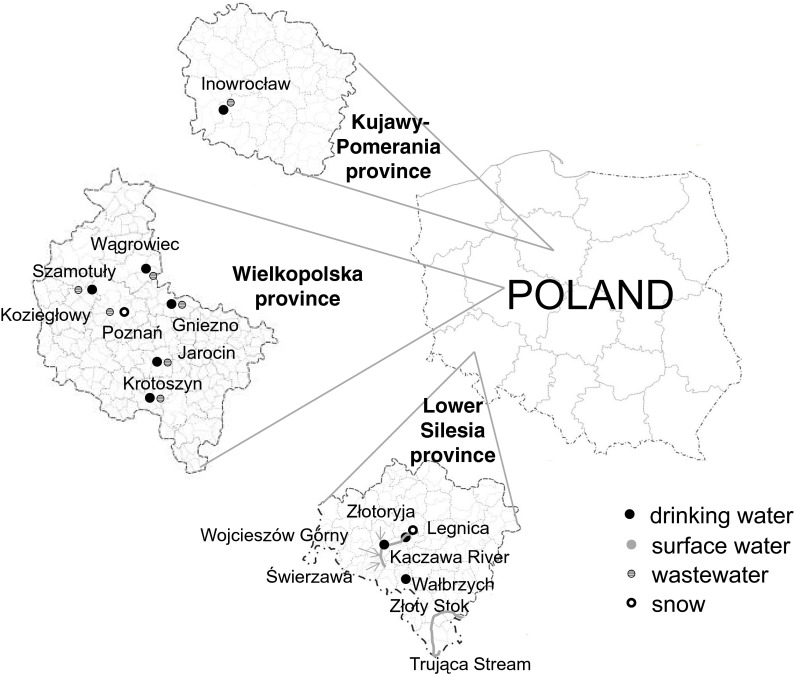
Sampling map of study area

Samples were collected in the area of Poland from provinces of Wielkopolska, Kujawy-Pomerania, and Lower Silesia. Sample collection locations are presented in detail on the sampling map of study area (Fig. 1). One sample at each sampling point was collected. In total, 23 samples of drinking water, surface water, wastewater, and snow were submitted to analysis, three replicate measurements of each were made.

#### Drinking water and wastewater

Drinking water and wastewater samples were collected from places located in three different provinces of Poland: Wielkopolska, Kujawy-Pomerania, and Lower Silesia. Samples were collected at points of water intake, from water treatment stations or municipal sewage treatment plants.

#### Surface water

Surface water samples were collected from Lower Silesia province, which is the region definitely much richer in arsenic than Wielkopolska or Kujawy-Pomerania. Samples were taken from the Kaczawa River and the Trująca Stream from few different locations.

##### Kaczawa River

Kaczawa is the left-bank tributary of Odra River. Water supply point of drinking water for the city of Legnica is situated on the Kaczawa River, in Smokowice. Upper course of the Kaczawa River flows through farmlands; its lower and middle course flows through industrially-agricultural areas, which makes the river exposed to impurities flowing from the farmlands and cultivated fields. Supplies of the Kaczawa River may also be a great source of contamination (RRIEP 2006).

##### Trująca Stream

The Nysa Kłodzka River is the largest river of the Kłodzko Basin, and it starts in the Lower Silesia province. Nysa Kłodzka River enters Lower Silesia province below a confluence point with an outlet of the Trująca Stream and above the Otmuchowski Reservoir. The Nysa Kłodzka River in its upper course flows through mountainous areas, functioning mainly as a tourist area. The river and its supplies collect the water from nature preservation areas as well as from farm areas. There are industrial plants working in the river catchment area (RRIEP 2006).

#### Snow

Snow samples were collected in the city of Legnica situated in the Lower Silesia province and in the city of Poznań situated in the Wielkopolska province. The Lower Silesia province is an area recognized as significantly richer in arsenic in comparison to other provinces. During its history, a gold mine was operating in Złoty Stok City (Lower Silesia). Apart from gold mining, mine also produced an arsenic trioxide from the local ore (www.kopalniazlota.pl/pl/historia/historia-kopalni-zlota). Aforementioned sample was collected near the copper smelter and the refinery located near city of Legnica. Second sample of snow, collected from the center of Poznań, may be treated as a control sample.

Sample collection and preparation.

All samples were collected in polyethylene bottles previously cleaned with metal-free detergent, rinsed with deionized water, then soaked in 5 % nitric acid for 24 h and finally rinsed once again with deionized water. After collection, samples were promptly transported in a cool-box to the laboratory. Total arsenic and arsenic species determination were followed by water quality parameter’s investigation (pH, conductivity, COD, BOD, TOC) and determination of major inorganic ions (*Ca*^2+^, *Mg*^2+^, *Na*^+^, *K*^+^, *Cl*^−^, $S{O}_4^{2-}$, $P{O}_4^{3-}$, $N{O}_3^{-}$) concentration. Results regarding water quality parameters and basic anions and cations were partially provided by water treatment stations or municipal sewage treatment plants, from where some of the samples had been collected. For measurements aiming at the determination of total arsenic concentration, samples were acidified with 125 μL of nitric acid (suprapur nitric acid of 65 % (*v*/*v*)) on each 125 mL of sample. However, for arsenic speciation analysis samples were immediately frozen and thawed just before the analysis. Samples were then filtered through syringe filters with a pore size of 0.45 μm. The snow samples (Lower Silesia province and Wielkopolska province) were collected to the polyethylene bottles with use of polyethylene spatula. They were taken from the surface and 2 cm deep into the snow layer, so not only the dark layer of the atmospheric dust but also clean snow underneath was collected. After the sample melted, the water which arose from the melting snow was sampled.

### Instrumentation

ELAN DRC II ICP-MS (PerkinElmerSCIEX, Ontario, Canada) was used for total arsenic determination as well as for arsenic species determination. In case of speciation analysis, ICP-MS was hyphenated with HPLC system. To collect the data, ions of arsenic at mass to charge ratio (*m*/*z*^+^) 75 were monitored. HPLC system consisted of a Perkin Elmer Series 200 HPLC Pump, Perkin Elmer Series 225 HPLC Autosampler and a Perkin Elmer Series 200 Column Oven. Autosampler was equipped with a Peltier Cooling Tray in order to keep the sample temperature equal to 4 °C. The anion exchange HPLC column PRP-X100 (4.6 × 150 mm) was used for arsenic species separation. The column was packed with 5 μm particles of styrene divinylbenzene copolymer with trimethylammonium exchange sites in PEEK hardware (Hamilton Company, Bonaduz, Switzerland). Applied column enabled to separate five arsenic species in 7 min using optimized HPLC conditions. Arsenic species eluted in the following order: AsB, As(III), DMA, MMA, and As(V).

The outlet of the HPLC column was connected via a switch, directly to the nebulizer of the ICP mass spectrometer. The data were collected using Chromera software (PerkinElmerSCIEX, Ontario, Canada). Operating conditions and optimal values of HPLC and ICP-MS parameters are presented in Table 1.

**Table 1:** Operating conditions for HPLC and ICP-MS systems

Parameter	Setting
HPLC	
Instrument	PE Series 200 HPLC Pump, PE Series 225 HPLC Autosampler and PE Series 200 Column Oven
Column	Hamilton PRP-X100
Elution	Isocratic
Mobile phase	Ammonium dihydrogen phosphate, ammonium nitrate
Concentration of mobile phase	0.01 mol
pH	9.2 ± 0.1
Flow rate	1.0 mL min^−1^
Injection volume	75 μL
Column temperature	25 °C
ICP-MS	
Instrument	PE Sciex ELAN 6100 DRC II
RF power	1250 W
Nebulizer gas flow	0.95 L min^−1^
Auxiliary gas flow	1.375 L min^−1^
Plasma gas flow	14.5 L min^−1^
Sampler and skimmer cones	Pt
Lens voltage	9.75 V
Detector mode	Dual (pulse counting and analog mode)
Data collection mode	^75^As^+^
Scan mode	Peak hopping
Dwell time	250 ms
Sweeps	1
Reading	2362

Total arsenic concentration was determined according to ISO 17294–2 (2003) standard; however, concentration of arsenic species was determined according to methodology developed and fully validated in our laboratory (Komorowicz and Baralkiewicz 2014).

### Reagents

**Fig. 2:**
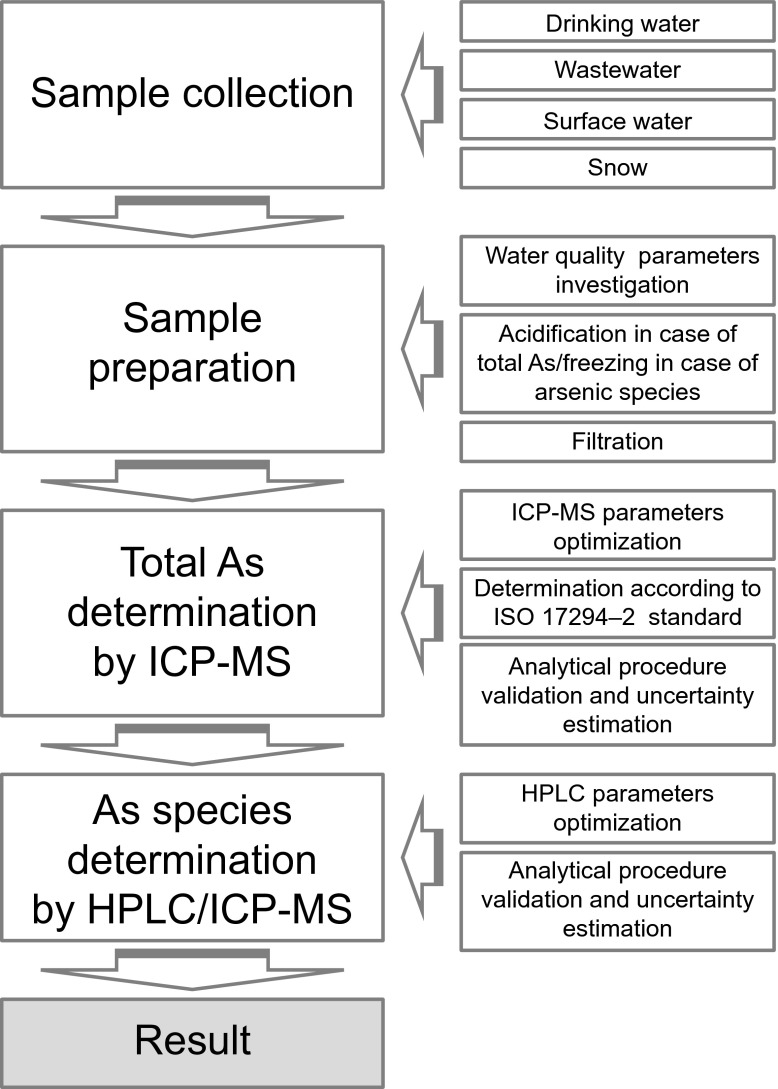
Diagram presenting the steps of analytical procedures

Chemicals and reagents used for total arsenic solutions and arsenic species (inorganic: arsenite (As(III)), and arsenate (As(V)); organic: arsenobetaine (AsB), monomethylarsenic acid (MMA), and dimethylarsenic acid (DMA)) solutions preparation, salts used for mobile phase preparation, buffer solution, reagents used for pH adjustment, as well as other chemicals used throughout the experiment, were described in details in the previously published work (Komorowicz and Baralkiewicz 2014). Particular steps of both analytical procedures—for total arsenic determination and for arsenic species determination—are presented on the Fig. 2 in the form of diagram.

### Figures of merit

A lot of information in the scientific field is made on the basis of analytical measurements. Ensuring that obtained analytical results are reliable is extremely important therefore the process of quality control is a significant step of each analytical procedure (Konieczka and Namieśnik 2010). In our work, the analytical procedures, for total arsenic determination by ICP-MS technique and arsenic species determination by HPLC/ICP-MS technique, were characterized by the following parameters: selectivity, linearity, limit of detection, limit of quantification, precision, and trueness. Traceability was assured by analysis of certified reference material and analysis of spiked samples, for total arsenic and arsenic species, respectively. Measurement uncertainty of the analytical result for total arsenic and for each arsenic species was assessed by the modeling approach according to the Guide to the Expression of Uncertainty of Measurement (GUM 1993; Konieczka and Namieśnik 2010). Validation parameters together with uncertainty for total arsenic and arsenic species are presented in Table 2.

**Table 2:** Characteristics of the analytical procedures of total arsenic determination and five arsenic species determination in water by ICP-MS and HPLC/ICP-MS, respectively

Analytical procedure parameters	Measurement result					
	Total As	AsB	As(III)	DMA	MMA	As(V)
Retention time (min)	–	1.7	2.1	2.4	4.2	6.3
Linear range (μg L^−1^)	0.2–20.0	0.2–10.0	0.2–10.0	0.2–10.0	0.2–10.0	0.2–10.0
Correlation coefficient	0.9999	0.9997	0.9998	0.9999	0.9998	0.9998
*LOD* (μg L^−1^)^a^	0.069	0.074	0.074	0.070	0.13	0.11
*LOQ* (μg L^−1^)^b^	0.21	0.22	0.22	0.21	0.39	0.33
Precision/retention time (%, CV)	–	0.53	0.56	0.68	0.69	0.21
Precision/concentration (%, CV)	1.7	2.4	2.0	1.6	2.3	1.6
Recovery (%)	101	99	100	100	98	101
Uncertainty (%)	7.1	12.0	13.0	5.6	9.6	8.6

Parameters were calculated as a mean value from ten replicated measurements

^a^ *LOD* was calculated as three times the SD from the blank samples (*n* = 10) with the addition of the arsenic concentration, which was close to the expected *LOD* value

^b^ *LOQ* values were calculated as three times the appropriate *LOD* values

#### Total arsenic

Total arsenic concentration was determined with ICP-MS technique according to validated methodology described in ISO 17294–2 (2003) standard. The calibration curve for total arsenic was constructed at a concentration range of 0.2–20.0 μg L^−1^. In order to verify the linearity of calibration curves, approach based on drawing a graph of constant response for *y*/*x* values (where *y* denotes the instrumental signal and *x* is the concentration of analytes in the standard solution) with acceptable deviation within ±5 % was applied. The chosen operating range for each of the calibration curves was statistically verified by checking the homogeneity of variances using Snedecor’s *F* test. Precision calculated by analysis of CRM of river water SLRS-5 (National Research Council Canada, Ontario, Canada) amounted to 1.7 % (*n* = 10). The traceability for the total arsenic measurement was assessed by determining the % bias between the measured concentration (*n* = 10) and certified value of the mentioned certified reference material. Obtained value was equal to 0.4176 ± 0.0071 μg L^−1^; however, certified value amounted to 0.413 ± 0.039 μg L^−1^. Calculated recovery was equal to 101.1 ± 1.7 %, which confirms that sample matrix influence is negligible. Combined uncertainty of total arsenic was estimated taking into consideration the following sources of uncertainty: measurement repeatability, calibration, and recovery. Expanded uncertainty (*U* [%], *k* = 2) estimated for total arsenic using “bottom-up” approach was equal to 7.1 %. It may be applied to the concentration range of 0.2–20.0 μg L^−1^.

#### Arsenic species

Arsenic species concentration was determined by commercially available HPLC/ICP-MS technique according to developed and fully validated in our laboratory methodology described previously (ISO/IEC 2005; Komorowicz and Baralkiewicz 2014). “In-house” traceability defined as trueness was assured by analysis of spiked samples. Uncertainty budget for each arsenic species was estimated according to the bottom-up approach (GUM 1993).

## Results and discussion

### Total arsenic concentration in water media of Poland

Table 3 presents the concentration of TAs and speciated arsenic in drinking water, surface water, wastewater, and snow in provinces of Wielkopolska, Kujawy-Pomerania, and Lower Silesia. Results obtained for drinking water samples confirmed that total arsenic level was significantly lower than 10 μg L^−1^ which is the maximum permissible concentration of arsenic in drinking water, according to guidelines of the World Health Organization (WHO 2011), the US Environmental Protection Agency (USEPA 2009) and European Union (Official Journal of the EU 1998). In the majority of the drinking water samples, collected from regions of Wielkopolska, Kujawy-Pomerania, as well as from the area of Lower Silesia, concentration of total arsenic was significantly below 1 μg L^−1^. The highest concentration of total arsenic was found in a sample collected in Wałbrzych—0.249 ± 0.018 μg L^−1^.

**Table 3:** Concentration of total arsenic and arsenic species in drinking water, surface water, wastewater, and snow samples presented with extended uncertainty (*n* = 3)

	Samples	Spike (μg L^−1^)	Concentration of arsenic species (c ± U) (μg L^−1^) (*k* = 2)										Concentration of TAs (c ± U) (μg L^−1^) (*k* = 2)
			RS	SS	RS	SS	RS	SS	RS	SS	RS	SS	
			AsB		As(III)		DMA		MMA		As(V)		
Drinking water													
1	Szamotuły	0.25	< LOD	0.242 ± 0.029	< LOD	0.260 ± 0.034	< LOD	0.254 ± 0.014	< LOD	0.245 ± 0.024	0.241 ± 0.021	0.463 ± 0.040	0.236 ± 0.017
2	Inowrocław	1.0	< LOD	0.97 ± 0.12	< LOD	0.98 ± 0.13	< LOD	1.011 ± 0.057	< LOD	0.983 ± 0.094	1.052 ± 0.090	2.10 ± 0.18	1.010 ± 0.072
3	Gniezno	0.5	< LOD	0.480 ± 0.058	< LOD	0.511 ± 0.066	< LOD	0.512 ± 0.029	< LOD	0.491 ± 0.047	0.451 ± 0.039	0.971 ± 0.084	0.435 ± 0.031
4	Jarocin	0.25	< LOD	0.251 ± 0.030	0.121 ± 0.016	0.340 ± 0.044	< LOD	0.262 ± 0.015	< LOD	0.262 ± 0.025	0.433 ± 0.037	0.690 ± 0.059	0.525 ± 0.037
5	Wągrowiec	0.25	< LOD	0.245 ± 0.029	< LOD	0.240 ± 0.031	< LOD	0.253 ± 0.014	< LOD	0.262 ± 0.025	< LOD	0.263 ± 0.023	< LOD
6	Krotoszyn	0.25	< LOD	0.262 ± 0.031	< LOD	0.252 ± 0.033	< LOD	0.250 ± 0.014	< LOD	0.260 ± 0.025	0.192 ± 0.016	0.452 ± 0.039	0.180 ± 0.013
7	Złotoryja	0.25	< LOD	0.231 ± 0.028	< LOD	0.261 ± 0.034	< LOD	0.242 ± 0.014	< LOD	0.241 ± 0.023	0.152 ± 0.013	0.381 ± 0.033	0.141 ± 0.010
8	Legnica	0.25	< LOD	0.253 ± 0.030	< LOD	0.250 ± 0.033	< LOD	0.262 ± 0.015	< LOD	0.274 ± 0.026	0.1024 ± 0.0088	0.370 ± 0.0329	0.1210 ± 0.0087
9	Wałbrzych	0.25	< LOD	0.241 ± 0.029	< LOD	0.250 ± 0.033	< LOD	0.241 ± 0.013	< LOD	0.244 ± 0.023	0.263 ± 0.023	0.501 ± 0.043	0.249 ± 0.018
Surface water													
10	Kaczawa River (Wojciechów Górny)	1.0	< LOD	1.02 ± 0.12	0.540 ± 0.070	1.58 ± 0.21	< LOD	0.982 ± 0.055	< LOD	0.973 ± 0.093	0.959 ± 0.082	2.02 ± 0.17	1.53 ± 0.11
11	Kaczawa River—above Świerzawa	2.0	< LOD	1.97 ± 0.24	0.191 ± 0.025	2.09 ± 0.27	< LOD	2.02 ± 0.11	< LOD	1.96 ± 0.19	2.57 ± 0.22	4.49 ± 0.39	2.840 ± 0.202
12	Kaczawa River—water intake for Legnica	1.0	< LOD	0.99 ± 0.12	< LOD	1.04 ± 0.14	< LOD	1.006 ± 0.056	< LOD	0.971 ± 0.093	0.971 ± 0.084	1.92 ± 0.17	0.928 ± 0.066
13	Trująca River—before wastewater inlet	5.0	< LOD	4.96 ± 0.60	0.342 ± 0.044	5.17 ± 0.67	< LOD	5.03 ± 0.28	< LOD	5.05 ± 0.48	10.19 ± 0.88	15.0 ± 1.3	10.94 ± 0.78
14	Trująca River—after wastewater inlet	20.0	< LOD	21.3 ± 2.6	< LOD	18.4 ± 2.4	< LOD	19.5 ± 1.1	< LOD	18.7 ± 1.8	3775 ± 324	58.4 ± 5.0	3778 ± 268
Wastewater													
15	Inowrocław	0.5	< LOD	0.513 ± 0.062	< LOD	0.521 ± 0.068	< LOD	0.503 ± 0.028	< LOD	0.485 ± 0.047	0.370 ± 0.032	0.908 ± 0.078	0.351 ± 0.025
16	Krotoszyn	0.25	< LOD	0.249 ± 0.030	0.153 ± 0.020	0.422 ± 0.055	< LOD	0.258 ± 0.014	< LOD	0.263 ± 0.025	0.2410 ± 0.021	0.456 ± 0.039	0.363 ± 0.026
17	Szamotuły	1.0	< LOD	1.02 ± 0.12	< LOD	1.010 ± 0.13	< LOD	1.009 ± 0.057	< LOD	0.978 ± 0.094	0.942 ± 0.081	1.98 ± 0.17	0.1020 ± 0.0072
18	Jarocin	0.25	< LOD	0.228 ± 0.027	0.111 ± 0.014	0.333 ± 0.043	< LOD	0.249 ± 0.014	< LOD	0.257 ± 0.025	0.231 ± 0.020	0.503 ± 0.043	0.324 ± 0.023
19	Wągrowiec	0.25	< LOD	0.248 ± 0.030	< LOD	0.242 ± 0.031	< LOD	0.251 ± 0.014	< LOD	0.241 ± 0.023	0.162 ± 0.014	0.402 ± 0.035	0.150 ± 0.011
20	Gniezno	1.0	< LOD	0.98 ± 0.12	< LOD	0.98 ± 0.13	< LOD	0.993 ± 0.056	< LOD	0.981 ± 0.094	0.782 ± 0.067	1.81 ± 0.16	0.780 ± 0.074
21	Koziegłowy	1.0	< LOD	0.97 ± 0.12	0.416 ± 0.054	1.48 ± 0.19	< LOD	1.014 ± 0.057	0.281 ± 0.027	1.26 ± 0.12	1.18 ± 0.10	2.23 ± 0.19	1.82 ± 0.13
Snow													
22	Legnica	20.0	< LOD	20.8 ± 2.5	81 ± 11	42.0 ± 5.5	< LOD	19.6 ± 1.1	< LOD	19.5 ± 1.9	1.81 ± 0.16	22.4 ± 1.9	83.1 ± 5.9
23	Poznań	0.25	< LOD	0.242 ± 0.029	< LOD	0.255 ± 0.033	< LOD	0.252 ± 0.014	< LOD	0.247 ± 0.024	< LOD	0.256 ± 0.022	< LOD

LOD values: 0.074 μg L^−1^ for AsB, 0.074 μg L^−1^ for As(III), 0.070 μg L^−1^ for DMA, 0.13 μg L^−1^ for MMA, and 0.11 μg L^−1^ for As(V); certified value of total As for CRM SLRS-5—0.413 ± 0.039 μg L^−1^, obtained value—0.4176 ± 0.0071 μg L^−1^

*RS* real sample, SS spiked sample

Surface water samples were collected from the Kaczawa River and the Trująca Stream situated in the Lower Silesia province. Samples from the Kaczawa River were taken at three different points (from Wojcieszów Górny; above Świerzawa City; from water intake for Legnica city). Obtained results of total arsenic concentration for mentioned samples ranged from 0.928 ± 0.066 to 2.84 ± 0.20 μg L^−1^. However, total arsenic concentration in Trująca Stream was found to be much higher than these determined in Kaczawa River. In this case, samples were collected at two points. The first sample was collected from the upper reaches of the Trująca Stream, just above the Złoty Stok City. Second sample was taken close to the bridge located on the Błotnica-Topola road, at the close of a uniform water body. Obtained results were as follows: 10.94 ± 0.78 and 3778 ± 268 μg L^−1^. Such a high concentration of total arsenic in the second sample from Trująca Stream must be caused by the local geochemical structure, which includes deposits of arsenic. Złote Góry massif, within Trująca Stream is located, is mostly composed of mica slates of Proterozoic era relatively of the Lower Cambrian. Among them, there are slots of crystalline dolomite limestone with arsenic ores. In this area, the contact deposit with intrusive-hydrothermal character appears. Changes of dolomitic limestone in fine-grained diopsidic rocks through supplying the silica occurred during neighboring syenite intrusion. Afterward, diopsidic arsenic and gold rocks were introduced by hydrothermal silica. This process occurred fundamentally in contact rocks (http://zlotystok.salwach.pl/przemysl/wydobycie_rud_arsenu). Deposits of arsenic were primarily exploited in the area of Złoty Stok. While exploitation of arsenic ores, another deposit was found—gold. Gold, associated with the occurrence of arsenic ore, was most likely discovered in the seventh century, and the oldest record of mining operations conducted here from the thirteenth century. It is thus the oldest gold mine in Poland, where the exploitation of ore arsenic and recovery of gold continued until 1962 (http://www.kopalniazlota.pl/pl/historia/historia-wydobycia-zlota). According to opinion placed in the report about quality of rivers of Lower Silesia province prepared by Regional Inspectorate of Environmental Protection in Wrocław, the wastewater from mechanical-biological sewage treatment plant in Złoty Stok City, as well as from mechanical-biological sewage treatment plant of Material and Paint Plant is released to the Trująca Stream (RRIEP 2006, RIEPW 2001). However, in the plants mentioned above the wastewater comes under many processes and after sedimentation of deposit in the sedimentation tank refined wastewater is received. Obtained wastewater has reduced content of contaminants by approximately 90 to 96 %, and in this form, it gets to the channel which drains it to the Trująca Stream. Hence, the wastewater introduced to the Trująca Stream could not have considerable impact on increase of arsenic concentration in its water, so high concentrations of arsenic in this region are due to its geogenic characteristics.

Total arsenic concentration in the wastewater samples collected in the Wielkopolska and Kujawy-Pomerania was comparable with results obtained for drinking water samples. The highest concentration was determined in a sample collected from the wastewater treatment plant in Koziegłowy in which concentration of this element was equal to 1.82 ± 0.13 μg L^−1^.

**Fig. 3:**
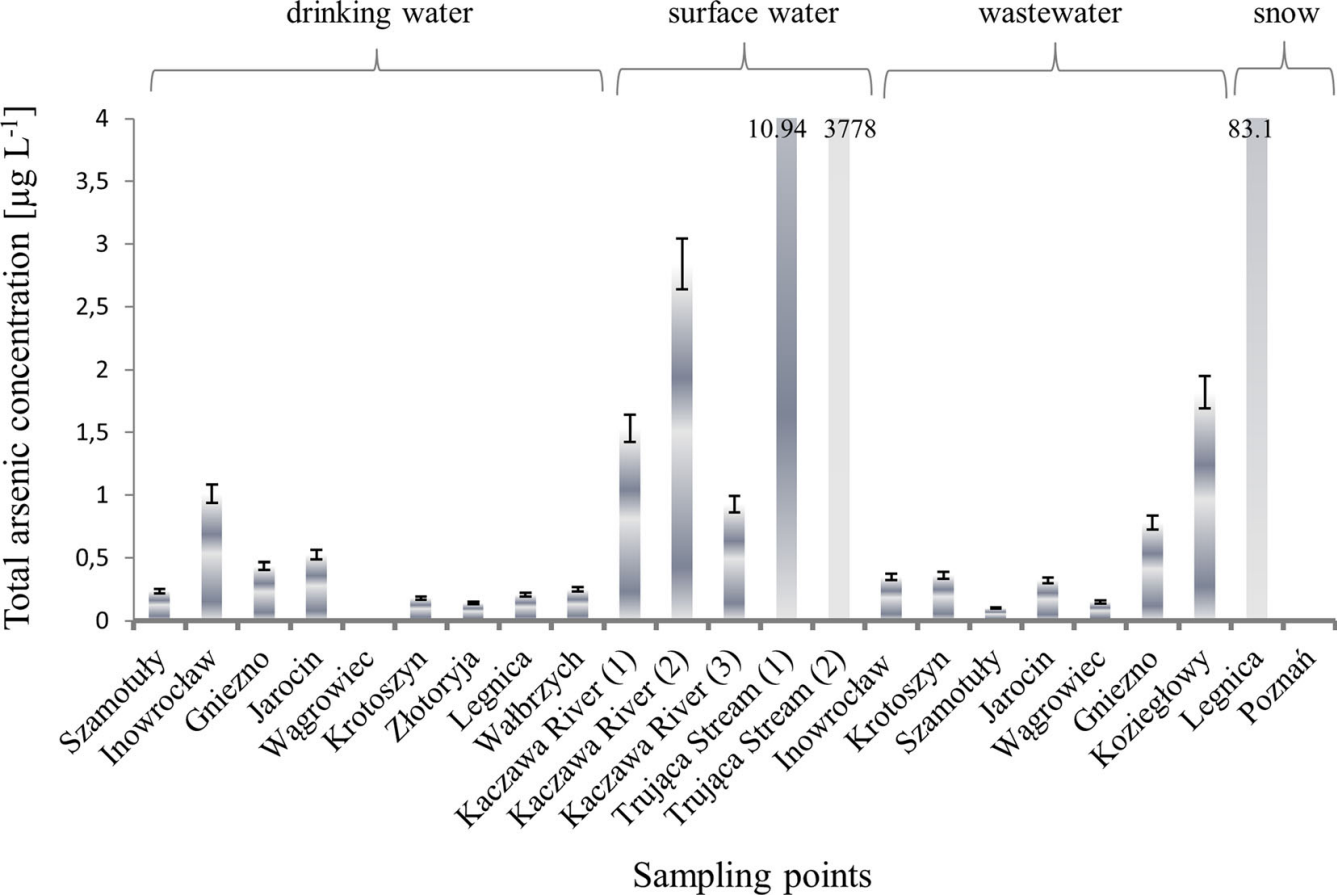
Total arsenic concentration in different sample types determined by ICP-MS method

Snow samples from two provinces were submitted to analysis. The first one was collected in the city of Legnica situated in Lower Silesia province. Sampling point was situated near the copper smelter and the refinery. Sample was characterized by a dark layer of atmospheric dust localized on its surface. Analysis revealed high concentration of arsenic equal to 83.1 ± 5.9 μg L^−1^. Almost all of arsenic in mentioned sample (97 %) was in the form of As(III), the most toxic arsenic species. Such a high concentration of arsenic could only come from particulate matter suspended in the air in Legnica, where, according to the report of the Regional Inspectorate for Environmental Protection in Wroclaw (RRIEP 2011), total arsenic concentration is the highest in comparison to other cities of Lower Silesia. High annual concentration of arsenic (5.9 ng m^−3^) but still placing within the permissible contamination level was observed only in Legnica. However, further potential increase only by 2 % would have caused exceeding permissible contamination level of arsenic (RRIEP 2011). At the same time, the second snow sample was collected in the centre of Poznań (the largest city in Wielkopolska province). Appearance of this sample was very similar to that collected in Legnica, its surface was also covered by dark atmospheric dust. However, the concentration of arsenic in the sample was below the limit of detection. Total arsenic concentration in all described samples is shown in Fig. 3.

### Arsenic species concentration in water media of Poland

Samples dedicated to speciation analysis were analyzed directly; however, in the majority of samples, concentration of some species was below the limit of detection. We calculated the recovery values for each arsenic species by analysis of spiked samples due to lack of certified reference materials having certified concentration of arsenic species. Spike concentrations as well as the results of AsB, As(III), DMA, MMA, and As(V) determination in drinking water, surface water, wastewater, and snow are presented in Table 3 as an average of three measurements together with the expanded uncertainty.

From among species of our interest, only As(V) was present in all of the investigated samples. In more than 60 % of samples, As(V) was the only one occurring arsenic species. In two of the investigated samples, drinking water and snow ones collected in Wielkopolska, arsenic was not detected. In 35 % of samples, we found As(III), the most toxic arsenic species. The organic compound, MMA, was present in only one of all the investigated samples. Other organic compounds such as DMA and AsB were not detected in any sample, which may indicate a lack of the organic compounds responsible for methylation of inorganic species to organic forms.

### Total and speciated arsenic concentration in the world

Table 4 demonstrates the results obtained for total arsenic and arsenic species in aqueous samples collected from many different regions in the world as well as the results obtained in this study. Besides concentration of arsenic and its species which is the crucial information, many other important issues such as sample sites and matrix, geogenic characteristics, sample pretreatment, and analytical techniques used are gathered in mentioned table. Majority of worldwide reports concerns groundwater samples as it often constitutes the primary source of drinking water. There are a lot of countries, such as: Vietnam, Pakistan, China, Argentina, Italy, India, Bangladesh, and Mongolia where reported concentration of arsenic amounts to even more than thousands of μg L^−1^ which is alarming (Berg et al. 2001; Brahman et al. 2013; Shakoor et al. 2015; Guo and Wang 2005; Smedley et al. 2002; Aiuppa et al. 2003; Rahman et al. 2015; Gong et al. 2006). Results regarding drinking water (Musil et al. 2014) are similar to these obtained in this work, significantly lower than 10 μg L^−1^ which is the maximum permissible concentration of arsenic in drinking water. In case of surface water samples, different levels of arsenic may be distinguished, which is also very similar to the results obtained by us. Starting from relatively low values not exceeding 20 μg L^−1^ (Baig et al. 2010) through hundreds of μg L^−1^ (Torrance et al. 2012) to these extremely high, determined exemplary in Argentina (up to 4780 μg L^−1^) (Gammons et al. 2005; Farnfield et al. 2012). The highest values are at the same level as result obtained by authors for surface water collected from Trująca Stream (up to 3778 μg L^−1^).

**Table 4:** Concentration of total arsenic and its species in different types of water samples—selected examples from the world

Sampling sites	Geogenic characteristics	Matrix	Total arsenic (μg L^−1^)	Arsenic species (μg L^−1^)	Sample pretreatment	Analytical techniques	References
Southeast of Tianjin, China	No data	River water Lake water	2.98 ± 0.10 0.56 ± 0.13	As(V) 2.75 ± 0.12 –	Filtration through a 0.22 μm microporous membrane; TAs digestion with use of concentrated HCl and HNO_3_ in a teflon digestion bomb; digested sample was mixed with thiourea, HCl and deionized water.	LC-HG-AFS	Yu et al. (2014)
Kolkata city, West Bengal, India	No data	Groundwater	0.244–1.29	As(V) 0.42 ± 0.02 As(III) 0.80 ± 0.02	Na data	HPLC INAA	Acharya et al. (2009)
Fourteen peri-urban communities located in-land and along the coast of the Gulf of Guinea in the Sekondi-Takoradi Metropolis, Ghana	The study area is underlain by rocks formed over two geochemical periods, they contain tectonic domain of volcano-plutonic group (volcanic belts) and a sub-domain of synvolcanic intrusive rock. The volcanic belts predominantly consist of metamorphosed tholeiitic lavas, minor volcaniclastics, acidic composition, and “belt-type” granitoids. The early Paleozoic Sekondian group consists of tectonic domain of sedimentary basin. It is composed of sandstone and interbedded shale. The sedimentary basins are composed of volcaniclastics, wakes, and argillites which are intruded by aluminum granite plutons metamorphosed to amphibolite facies.	Groundwater	<0.002–136	–	Filtration through a 0.45-μm pore size cellulose acetate membrane. After filtration, water samples were acidified with 65 % trace metal-grade nitric acid solution to a pH <2.	HG-AAS	Affum et al. (2015)
Samples purchased from a local store in Prague	No data	Drinking water	0.409 ± 0.011 0.269 ± 0.011	As(V) + As(III) 0.409 ± 0.02 As(V) + As(III) 0.260 ± 0.011 MMA 0.0015 ± 0.0005 DMA 0.0073 ± 0.0013 TMAO 0.0007 ± 0.0022	Samples were measured directly for As(III) and TMAO determination, for As(III + V), MMA, and DMA determination, 2 % (*m*/*v*) solid L-cys was added at least 1 h prior to analysis.	HG-CT-ICPMS HG-CT-AFS	Musil et al. (2014)
Northern China	No data	Spring water Well water	–	n.d. As(III) up to 1.4 As(V) up to 7.5 As(III) up to 1.5	SPE procedure was used for the preconcentration and separation of As(III) and As(V).	SPE WDXRF	Hagiwara et al. (2015)
Wuhan, China (rainwater); central part of East Lake and Yangtze River, Wuhan, China (lake and river water)	No data	Rain water	–	As(V) 0.64 ± 0.02 As(III) 0.22 ± 0.01	Filtration through a 0.45 μm membrane.	SPE-ICP-MS	Peng et al. (2015)
		River water	–	As(V) 2.32 ± 0.11 As(III) 0.43 ± 0.05			
		Lake water	–	As(V) 2.12 ± 0.11 As(III) 0.40 ± 0.03			
Kermanshah, Iran	No data	Tap water Lake water Well water	0.093 ± 0.0035 0.058 ± 0.0045 n.d.	As(V) 0.055 ± 0.0020 As(III) 0.038 ± 0.0015 As(V) 0.033 ± 0.0025 As(III) 0.025 ± 0.0020 n.d.	Extraction: SPE coupled with DLLME-SFO method using DDTP as a proper chelating, used as an ultra preconcentration technique.	SPE-DLLME-SFO GF-AAS	Shamsipur et al. (2014)
Sarkisla Plain, Sivas/Turkey	Sarkisla Plain is a tectonic graben system deposited by the sedimentary units eroded from nearby geological rocks; the basement rock is Paleocene volcanic rocks mainly composed of dark colored pyroclastic andesite and basalt; they are overlaid by volcano-sedimentary rocks and limestone, gypsum, sandstone, claystone and mudstone layers; finally alluvial sediments overlie all these formations and are mostly composed of clayey sand, clayey gravel and gravel.	Well water Spring water	7–345.4 0.5–26.6	–	Filtration through a 0.45-μm filter; acidification with nitric acid in order to obtain a pH value of <2.	ICP-AES	Simsek (2013)
Hanoi, Vietnam	The Red River carries huge quantities of silt, rich in iron oxide, because of the large proportion of easily crumbled soil in its basin. Naturally anoxic conditions in the aquifers are due to peat deposits, and consequently, the groundwaters contain large amounts of iron and manganese that are removed in the Hanoi drinking water plants by aeration and sand filtration.	Groundwater	<1–3050	–	Acidification with 1 mL of concentrated nitric acid. The few turbid samples (i.e., less than 5 %) were filtered (0.45 μm) in the laboratory and acidified thereafter.	HG-AAS	Berg et al. (2001)
Khairpur Mir’s, Pakistan	The study areas are situated on the east bank of the Indus River, composed of quaternary alluvial-deltaic sediments derived from Himalayan rocks.	Surface waters: Canals Rivers Lakes Municipal treated water Groundwater Hand pumps	4.2–8.0 3.0–5.3 10.0–18.3 5.00–8.30 9.2–163 9.2–361	As(V) 2.0–4.4 As(III) 2.1–3.2 As(V) 1.1–2.2 As(III) 1.3–3.0 As(V) 2.35–12.07 As(III) 1.93–5.68 As(V) 2.70–4.66 As(III) 1.90–3.38 As(V) 5.6–77.19 As(III) 3.1–71.2 As(V) 5.90–238 As(III) 2.80–114	In case of TAs determination samples were pre-concentrated on an electric hot plate and filtered. The As(III) was isolated through adding the chelating agent then using the mechanical shaker and finally centrifugation.	SPE GF-AAS	Baig et al. (2010)
Different states in USA	No data	Drinking water wells	13.0–69.0	As(V) 0.0–100.0 As(III) 0.2–66.8	The sample’s handling consisted of a three-step process that required three arsenic analyses: (1) raw water sample, (2) filtered (0.45 μm) water sample, and (3) an anion resin treated water sample. Speciation kits, which included resin columns, 0.45-mm filters, and three sample bottles with preservatives, were prepared.	Anion exchange resin Dowex 1-X8 ICP-MS	Sorg et al. (2014)
The vicinity of Fallon, Nevada, the Republic of Bangladesh, near Golden, Colorado (groundwater); Mississippi and Arkansas (groundwater and surface water); Colorado (acid mine drainage samples)	Groundwater samples were collected from sites in the vicinity of Fallon, Nevada, sites in the Republic of Bangladesh, and sites near Golden, Colorado; surface water and groundwater samples were collected in Mississippi and Arkansas from agricultural areas that use organic arsenic-containing herbicides; acid mine drainage samples were collected at sites clustered within three mineralized regions of Colorado.	Groundwater, surface water, acid mine drainage samples	–	As(V) up to 3700 As(III) 13,000 MMA up to 100 DMA up to 10	Filtration through either a 0.45-μm pore-size syringe filter or an in-line capsule filter. EDTA was added immediately to all filtered samples, to preserve the distribution of arsenic species by chelating metal cations, buffering the sample pH, and reducing microbial activity.	HPLC-ICP-MS	Bednar et al. (2004)
Two sub districts of Tharparkar, Pakistan	The Tharparkar district is very rich in minerals resources like china clay, granite, coal, and salts. Geoelectric, drilling and geophysical log data indicate four major divisions of lithological sequences in the whole Thar Desert. These zones are sand dune, sub-recent deposits, coal-bearing formations of Paleocene, igneous and basement complex of Precambrian age.	Groundwater	6–4330	As(V) 4–2650 As(III) 2–1390	Filtration through 0.45-μm filter paper. In case of TAs determination samples were acidified with 2–3 drops of concentrated HNO_3_.	CPE (As(III)) SPE (iAs) ETAAS	Brahman et al. (2013)
Counties of Hungary	No data	Public well water	7.2 ± 0.2–210.3 ± 4.9	As(V) < 0.8–163.3 ± 6.5 As(III) < 0.1–44.5 ± 4.9	For TAs determination samples were acidified with 100 μL of cc. HNO_3_/100 mL sample, in case of arsenic species simple field separation method was applied by using solid-phase extraction anion exchange cartridges.	SPE ICP-SF-MS	Sugár et al. (2013)
Western Hetao Plain, northern China	Hetao Plain was located in a fault basin formed at the end of Jurassic with fine clastic sediments. They are overlaid by Tertiary red sandstone and shale with gypsum and rock salt. The muddy clay, silt, fine sand and interlayer of peat can be also distinguished. The Quaternary groundwater systems can be basically divided into two groups of aquifers: 1) alluvial and lacustrine deposits (clay, medium sand, fine sand, and silt); 2) lacustrine mud clay and fine sand. Groundwater is recharged by vertically infiltrating meteoric water, laterally flowing groundwater from bedrocks along Yin Mountains front, and by irrigation return flow and leakage from Yellow River in the south area.	Groundwater	76–1093	PAs 7.29–82.6 As(V) 4.6–81.6 As(III) up to about 1080 MMA and DMA < 2	Filtration (0.45 μm) on-site; acidification with use of 1 % *v*/*v* HNO_3_ in case of TAs. In case of arsenic speciation disposable syringes and 0.45-μm membrane filters, resin-based strong cation-exchange cartridge, and a silica-based strong anion exchange cartridge were used to separate PAs, As(III), As(V), MMA, and DMA species. Several samples were filtered and acidified with HCl to pH 1 for As speciation, using HPLC/ICP-MS.	HPLC-ICPMS HG-AFS	Deng et al. (2009)
Ba Men, Inner Mongolia	The areas of endemic arsenic poisoning, well watermay contain arsenic at concentrations in the hundreds of micrograms per liter. The arsenic in these wells is released from natural mineral deposits, and the well water is the primary drinking water source.	Well water	362.9–734.1	PAs 46.1–119.3 As(V) 126.7–175.4 As(III) 153.3–493.6	Filtration for soluble arsenic species, PAs was captured the filter and washed with acid to resolubilize the arsenic; acidification was used in case of TAs determination.	LC-HGAFS ICP-MS	Gong et al. (2006)
Murshidabad, West Bengal, India	No data	Tube-well water	13.1–618.3	As(V) 7.7–184.5 As(III) 6.8–501.7 MMA up to 2.1 DMA up to 0.7	The samples were split into two groups: (1) acidified with nitric acid to 0.1 % *v*/*v*; (2) unacidified. Acidified water samples were filtered through 0.45-μm membrane filters.	HPLC-ICPMS FI-ICPMS	Shraim et al. (2002)
Datong basin, northwestern China	Groundwater in the basin aquifers was basically recharged by infiltrating meteoric water and by laterally penetrating fracture water from basalt, metamorphic rocks, limestone, sandstone and shale along the mountain front around the Datong basin.	Groundwater	<0.1–1530.1	–	Filtration through 0.45-μm millipore filter paper and acidification.	ICP-MS	Guo and Wang (2005)
LaPampa, Argentina	The topmost part of the sequence consists of blanketing loess deposit (predominantly silts and fine sands); their mineralogy is dominated by plagioclase with variable amounts of quartz, alkali feldspar, often severely altered ferromagnesian minerals, pumice fragments, calcite and heavy minerals No discrete As minerals were identified petrographically.	Groundwater	<4–5300	As(III) < 3–110	Filtration (0.45 mm); in case of As(III) determination samples were acidified to pH 4 (HCl) and for determination of TAs to 2 % *v*/*v* (HCl).	HG-ICP-AES	Smedley et al. (2002)
Volcanic río Agrio and the geothermal waters of Copahue, Argentina	The waters of río Agrio (an acidic river) originate from the crater-lake of the active stratovolcano Copahue. The crater-lake waters are Cl–SO_4_ brines formed by the dissolution of magmatic gases. The addition of SO_2_ gas to water can cause a disproportionation reaction to occur generating acidity and solid sulfur. Subsequently the acid fluids react with the surrounding rocks and can acquire trace elements.	Surface water	<0.2–3783	As(V) < 0.02–19.5 As(III) < 0.2–347 MMA < 0.02–35.7 DMA < 0.02–10.1	Filtration through a 0.45-μm membrane filter; acidification using 0.1 mL concentrated nitric acid (15.8 M); the solid phase extraction (SPE) cartridges were used for arsenic species separation.	SPE ICP-MS	Farnfield et al. (2012)
The Lucky Shot Gold Mine in Hatcher Pass, south-central Alaska	The deposit at Lucky Shot is hosted within the quartz diorite pluton with fine-grained gold associated with sulfide pods within quartz veins. The presence of pyrite and arsenopyrite in the quartz veins gives rise to elevated levels of As in water draining the mine adits and tailings pile.	Surface water (water samples from streams, adits and boreholes around the mine)	0.97–752.5	As(V) up to 26.8 As(III) 0.37–725.7	Filtration using a 0.45-μm disposable filter. In case of TAs determination - acidification with 100 μL of ultra-pure concentrated nitric acid after filtration; in case of As species determination—filtration from the bulk sample into a pre-cleaned 50-mL HDPE tube, containing 100 μL of ultra-pure nitric acid.	LC ICP-MS	Torrance et al. (2012)
Patagonia, Argentina	Rio Agrio watershed owes its acidity to volcanic inputs of HCl, HF, and H_2_SO_4_ at the headwaters of the river. The presence of Fe minerals is observed. Copahue crater lake—with extremely high concentrations of Cl, SO_4_, and rock-forming elements; the chemistry of the lake is also influenced by direct precipitation and melting of summit glaciers and snowpack. Lake Caviahue—receives a large amount of runoff and overland flow during the Austral Spring due to snowmelt from the surrounding highlands.	Water—volcanically acidified watershed	Up to 4780	–	Filtration using membrane filters (pore size 0.45 μm); acidification (where needed) in the field laboratory to 5 % with grade nitric acid.	ICP-AES	Gammons et al. (2005)
Volcanic aquifers from southern Italy	In this paper aquatic geochemistry of arsenic is discussed on the basis of large number of papers; geogenic characteristic of sampling sites is described in particular paper.	Groundwater	0.1–6441	As(V) < 0.50–868 As(III) < 0.50–5570	–	HG-AFS	Aiuppa et al. (2003)
Pławniowice Reservoir, Poland	Pławniowice Reservoir belongs to the anthropogenic reservoirs and its water quality is mainly influenced by nutrients, organic compounds, heavy metals and suspensions introduced into the reservoir.	Bottom water	0.96–3.26	As(V) 0.18–1.49 As(III) 0.01–1.91	Acidification with spectral pure nitric acid; filtration through a 0.22-μm PES syringe filter in case of TAs.	HPLC-ICP-MS	Jabłońska-Czapla et al. (2014)
Rural areas (Chichawatni, Vehari, Rahim Yar Khan) of Punjab, Pakistan	Chichawatni city has a semi-arid alluvium plain area with the exception of a few belts of ravines and uneven land formed by gully erosion along the lower Bari Doab and its distributaries. Vehari consists of alluvium plain area with fertile land which is irrigated with the fertile water of Chenab and Ravi Rivers. Rahim Yar Khan city is divided into three main physical features: riverine area, canal irrigated area, and desert area (Cholistan).	Groundwater	41.5 ± 45.6–95.0 ± 60.5	Speciated As in selected groundwater samples: As(V) from 33 to 100 % As(III) from 0 to 67 %	Samples used to analyze TAs were acidified on-side by adding 2–3 drops of concentrated nitric acid. For As speciation, samples were preserved with 0.025 M EDTA.	IC-ICP-MS with ORS system	Shakoor et al. (2015)
Noakhali district, Bangladesh and West Bengal, India	The study area Noakhali is situated in flood plain region of Bangladesh. The deltaic plain and flood plains of the Ganga–Brahmaputra river system are the most As-contaminated areas in Bangladesh. The Bengal deltaic plain consists of sediments deposited by the Ganges, Brahmaputra, and Meghna rivers and their tributaries and distributaries. Arsenic in groundwater is hosted by the sediments deposited by the meandering river channels during the late Quaternary or the Holocene age.	Groundwater	1.5–587.6 (Bangladesh) 0.1–1219 (West Bengal, India)	–	Samples were preserved in 7 M nitric acid.	ICP-MS with ORS system	Rahman et al. (2015)
Shahpur block, Bhojpur district, Bihar state, India	No data	Groundwater	Up to 1805	–	One drop of nitric acid (7 M) was added for 10 ml of sample.	FI-HG-AAS	Chakraborti et al. (2016a)
Patna district (capital of Bihar) in the middle Ganga plain, India	No data	Groundwater	Up to 1466	–	One drop of nitric acid (1:1) was added for 10 ml of sample.	FI-HG-AAS	Chakraborti et al. (2016b)
Provinces of Wielkopolska, Kujawy-Pomerania and Lower Silesia, Poland	Surface water: Złote Góry massif, within Trująca Stream is located, is mostly composed of mica slates of Proterozoic era relatively of the Lower Cambrian. Among them there are slots of crystalline dolomite limestone with arsenic ores. In this area the contact deposit with intrusive-hydrothermal character appears. Changes of dolomitic limestone in fine-grained diopsidic rocks through supplying the silica occurred during neighboring syenite intrusion. Afterwards, diopsidic arsenic and gold rocks were introduced by hydrothermal silica.	Drinking water	Up to 1.010 ± 0.072	As(V) 1.052 ± 0.090 As(III) 0.121 ± 0.016	In case of TAs determination, samples were acidified with 125 μL of nitric acid (suprapure nitric acid of 65 % (*v*/*v*)) on each 125 mL of sample. All samples were filtered through syringe filters with a pore size of 0.45 μm.	HPLC/ICP-MS	This work
		Surface water	Up to 3778 ± 268	As(V) 3775 ± 324 As(III) 0.540 ± 0.070			
		Wastewater	Up to 1.82 ± 0.13	As(V) 1.18 ± 0.10 As(III) 0.416 ± 0.054 MMA 0.281 ± 0.027			
		Snow	Up to 83.1 ± 5.9	As(V) 1.81 ± 0.16 As(III) 80.8 ± 10.5			

## Conclusion

This study demonstrates the results of determination of total arsenic and arsenic species concentration in different types of water: drinking water, wastewater, surface water, and snow collected in Poland. The results point the following:

- Concentration of total arsenic in examined drinking water samples was in the range of 0.14–1.01 μg L^−1^. In all investigated samples, As(V) was the dominant form, in the majority, the only one occurring.
- High concentration of arsenic, up to 3778 μg L^−1^, was determined in surface water of the Trująca Stream. However, in samples collected from the Kaczawa River, situated in the same province as Trująca Stream, total arsenic concentration was in the range of 0.93–2.84 μg L^−1^. As(V) predominated, however As(III) was also present.
- Concentration of total arsenic in wastewater samples was comparable with those determined in drinking water samples and ranged from 0.10 to 1.82 μg L^−1^.
- Total arsenic concentration determined in a snow sample collected in Legnica was equal to 83.1 ± 5.9 μg L^−1^. More than 97 % of its concentration was confirmed to be As(III), the most toxic arsenic species. This high concentration was confirmed by the corresponding reports concerning air quality in Lower Silesia province.

Information relating total arsenic and its species concentration in different types of water samples collected in the area of Poland will help to broaden the knowledge in this field in the world, especially results regarding Trująca Stream which indicated high arsenic concentration caused by the local geochemical structure, which includes deposits of arsenic.

## Abbreviations

As(III): Arsenite
As(V): Arsenate
AsB: Arsenobetaine
CPE: Cloud point extraction
CRM: Certified reference material
DDTP: Diethyldithiphosphate
DMA: Dimethylarsenic acid (dimethylarsinate)
EDTA: Ethylenediaminetetraacetic acid
ETAAS: Electrothermal atomic absorption spectrometry
FI-HG-AAS: Flow injection-hydride generation atomic absorption spectrometry
FI-ICPMS: Flow injection inductively coupled plasma mass spectrometry
GF-AAS: Graphite furnace atomic absorption spectrometry
HG-AAS: Hydride generation atomic absorption spectrometry
HG-AFS: Hydride generation atomic fluorescence spectrometry
HG-CT-AFS: Hydride generation-cryotrapping coupled to atomic fluorescence spectrometry
HG-CT-ICPMS: Hydride generation-cryotrapping coupled to inductively coupled plasma mass spectrometry
HG-ICP-AES: Hydride generation inductively coupled plasma atomic/optical emission spectrometry
HPLC: High-performance liquid chromatography
HPLC/ICP-MS: High-performance liquid chromatography inductively coupled plasma mass spectrometry
iAs: Inorganic arsenic
ICP-AES: Inductively coupled plasma atomic emission spectrometry
ICP-MS: Inductively coupled plasma mass spectrometry
ICP-SF-MS: Inductively coupled plasma sector field mass spectrometry
INAA: Instrumental neutron activation analysis
LC: Liquid chromatography
LC-HGAFS: Liquid chromatography hydride generation atomic fluorescence spectrometry
MDL: Method detection limit
MMA: Monomethylarsenic acid (methylarsonate)
n.d.: Not detected
ORS: Octopole reaction system
PAs: Particulate arsenic
SPE: Solid-phase extraction
SPE-DLLME-SFO: Solid-phase extraction coupled with dispersive liquid–liquid microextraction based on the solidification of floating organic drop
TAs: Total arsenic
TMAO: Trimethylarsine oxide
USEPA: US Environmental Protection Agency
WDXRF: Wavelength-dispersive X-ray fluorescence spectrometry
WHO: World Health Organization
